# Presence of anti-acetylated peptide antibodies (AAPA) in inflammatory arthritis and other rheumatic diseases suggests discriminative diagnostic capacity towards early rheumatoid arthritis

**DOI:** 10.1177/1759720X211022533

**Published:** 2021-07-23

**Authors:** Paul Studenic, Alessia Alunno, Daniela Sieghart, Holger Bang, Daniel Aletaha, Stephan Blüml, Helmuth Haslacher, Josef S Smolen, Roberto Gerli, Günter Steiner

**Affiliations:** Division of Rheumatology, Department of Internal Medicine 3, Medical University of Vienna, Währinger Guertel 18-20, Vienna, 1090, Austria; Rheumatology Unit, Department of Medicine & Surgery, University of Perugia, Perugia, Italy; Division of Rheumatology, Department of Internal Medicine 3, Medical University of Vienna, Vienna, Austria & Ludwig Boltzmann Institute for Arthritis and Rehabilitation, Vienna, Austria; Orgentec Diagnostika GmbH, Mainz, Germany; Division of Rheumatology, Department of Internal Medicine 3, Medical University Vienna, Vienna, Austria; Division of Rheumatology, Department of Internal Medicine 3, Medical University Vienna, Vienna, Austria; Department of Laboratory Medicine, Medical University of Vienna, Vienna, Austria; Division of Rheumatology, Department of Internal Medicine 3, Medical University Vienna, Vienna, Austria; Rheumatology Unit, Department of Medicine & Surgery, University of Perugia, Perugia, Italy; Division of Rheumatology, Department of Internal Medicine 3, Medical University of Vienna, Vienna, Austria & Ludwig Boltzmann Institute for Arthritis and Rehabilitation, Vienna, Austria

**Keywords:** autoantibodies, diagnostic tests, inflammatory arthritis, rheumatoid arthritis, seronegative

## Abstract

**Aims:**

To determine the diagnostic value of anti-acetylated peptide antibodies (AAPA) in patients with rheumatoid arthritis (RA).

**Methods:**

Three acetylated peptides (ac-lysine, ac-lysine.inv and ac-ornithine) derived from vimentin were employed to measure AAPA by enzyme-linked immunosorbent assay (ELISA) in sera of 120 patients with early RA (eRA), 195 patients with established RA (est RA), 99 healthy controls (HC), and 216 patients with other inflammatory rheumatic diseases. A carbamylated and a citrullinated version of the vimentin peptide were used additionally. Receiver operating characteristics and logistic regression analyses were used to assess the discriminative capacity of AAPA.

**Results:**

AAPA were detected in 60% of eRA and 68.7% of estRA patients, 22.2% of HC, and 7.1– 30.6% of patients with other rheumatic diseases. Importantly, AAPA were also present in 40% of seronegative RA patients, while antibodies to the carbamylated peptide were detected less frequently. Diagnostic sensitivity of individual peptides for eRA was 28.3%, 35.8%, and 34% for ac-lysine, ac-ornithine, and ac-lysine.inv, respectively. Positive likelihood ratios (LR+) for eRA *versus* HC were 14.0, 7.1, and 2.1. While the presence of a single AAPA showed varying specificity (range: 84–98%), the presence of two AAPA increased specificity considerably since 26.7% of eRA, as compared with 6% of disease controls, were double positive. Thus, double positivity discriminated eRA from axial spondyloarthritis with a LR+ of 18.3. Remarkably, triple positivity was 100% specific for RA, being observed in 10% of eRA and 21.5% of estRA patients, even in the absence of RF and ACPA.

**Conclusion:**

AAPA are highly prevalent in early RA and occur also independently of RF and ACPA, thereby reducing the gap of seronegativity. Furthermore, multiple AAPA reactivity increased the specificity for RA, suggesting high diagnostic value of AAPA testing.

## Introduction

Rheumatoid arthritis (RA) is a chronic inflammatory and destructive musculoskeletal disease affecting synovial joints, leading to severe discomfort and disability.^1^ RA is characterized by the presence of autoantibodies that distinguish this disease from other types of inflammatory arthritis (IA) such as spondyloarthitis (SpA), psoriatic arthritis, or reactive arthritis. Among the numerous autoantibodies described in the literature, only two, rheumatoid factor (RF) and anti-citrullinated-peptide antibodies (ACPA), are currently employed in routine serodiagnostics. They are detectable in 50–70% of RA patients,^1,2^ and are included in the current classification criteria of RA.^3–6^ Autoantibodies can appear many years before disease onset, and both RF and ACPA have been associated with more severe disease, worse long-term outcomes,^7–9^ and a higher rate of relapses after withdrawal of disease modifying antirheumatic drug (DMARD) therapy. This evidence suggests that autoantibody positive (=seropositive) patients may be in greater need of immunosuppressive treatment.^10,11^

Since a significant proportion of patients test negative for RF and ACPA, the question remains whether seronegative RA may, in fact, represent a distinct disease subtype.^12^ This gap in seropositivity repeatedly fuels discussions on similarity of seronegative RA to seropositive RA and demands a closer diagnostic workup to differentiate it from other IA.^13–16^ During the last years, however, the identification of novel autoantibodies associated with RA, such as anti-carbamylated protein antibodies (anti-CarP) and antibodies targeting acetylated epitopes (anti-acetylated protein antibodies, AAPA) challenged the concept of seronegative RA, putting forward the hypothesis that the diagnostic gap can be closed or at least partially covered.^17–19^ Anti-CarP and AAPA are directed against epitopes containing post-translational modifications other than citrullination. Anti-CarP antibodies are directed at carbamylated residues, while AAPA recognize epitopes in which specific lysine residues have been modified enzymatically to carry an acetyl group. The acetylation of lysine residues occurs as a post-translational protein modification and is usually catalyzed by lysine acetyltransferase enzymes (KATs), but can also occur non-enzymatically, for example, in mitochondria or bacteria.^20,21^ Thus, AAPA are another member of the anti-modified protein autoantibody (AMPA) family.^22^

While anti-CarP antibodies may indeed have some value for RA diagnostics,^17,23^ the significance and diagnostic value of AAPA in RA has not been elucidated.^18^ To address this issue, we aimed to determine the prevalence of AAPA in patients with early RA or established RA, appropriate disease controls and healthy subjects, and thus derive their differential diagnostic value for RA *versus* other rheumatic condition. We focused on three differentially acetylated peptides derived from a vimentin peptide whose citrullinated isoform was shown to represent a major target of ACPA.

## Patients and methods

### Study design and patients

All samples used in this study had been previously acquired and stored in the biobank (biological specimen registry) of the Medical University of Vienna (EC-Number: 559/2005),^24^ and all patients had provided written informed consent, which follows the rules of the declaration of Helsinki and was approved by the Ethics Committee of the Medical University Vienna.^25^ Samples were drawn from 120 patients with early RA (eRA), 195 patients with established RA (est RA), 99 healthy subjects, 50 patients with SpA, and 166 patients with other rheumatic diseases including systemic lupus erythematosus (SLE), idiopathic inflammatory myopathies (IIM), granulomatosis with polyangiitis (GPA), and primary Sjögren’s syndrome (pSS). RA patients had been classified either by the ACR 1987 revised criteria,^26^ or the ACR/EULAR 2010 criteria,^6^ and were seen regularly approximately every 3–6 months, depending on the state of disease activity, by rheumatologists in the outpatient clinic of the Division of Rheumatology at the Medical University Vienna.^27^ Samples from patients with eRA were collected before initiation of treatment, in the majority of cases even before diagnosis of RA was confirmed. All patients with arthritis have been followed-up within “CARAbase” (Care of RA database) for a minimum time of 2 years, a clinical practice database for documentation of all clinical and therapeutic parameters.^28–30^ Patients with estRA (disease duration >3 years) were selected randomly from the database.

### Autoantibody testing

#### Acetylated peptides

AAPA IgG were measured by enzyme-linked immunosorbent assay (ELISA) as previously described using three acetylated peptides derived from a mutated form of the predominant vimentin epitope NH2-G**G**VYAT**R**SSAVR-OH.^10^ In this isoform, originally identified in synovial fluid of RA patients,^31^ the glycine residue in position 2 is replaced by arginine, resulting in NH2-G**R**VYAT**R**SSAVR-OH in which the arginine at position 7 (indicated as R, in bold) was changed to acetylated lysine (ac-lysine) or acetylated ornithine (ac-ornithine). In order to analyse the influence of neighboring amino acid residues acetylated lysine [G**-Lys(ac)-**VYAT**R**SSAVR] was introduced at the arginine in position 2 instead of position 7 (“inverse peptide”, ac-lysine.inv).^10^ The unmodified peptide served as negative control; sera reactive with both the modified and the control peptide were considered negative.^10^ Of note, very few sera were reactive with control peptides. In addition, antibodies to a citrullinated and a carbamylated version of the peptide were determined carrying the modification at position 7 as described previously.^10,18^

#### Precision and reproducibility of assays

Measurements of imprecision (inter-assay and intra-assay variability) were taken over four and six replicates, respectively. To assess the precision of the AAPA ELISA, low (L), medium (M) and high (H) value samples were assayed in five independent tests on 1 day (inter-assay) or in a single run (intra-assay). The intra-assay *coefficient of variation (*CV) was 8.3, 7.2, and 5.2% for the mean unit-values of 27.9, 155.4, and 803.3, respectively, whereas inter-assay CV was 4.2, 3.5, and 7.2% for mean unit-values as described before, respectively.

#### Standard curve

AAPA enzyme-linked immunosorbent assay (ELISAs) are considered to be semiquantitative because the readouts from experimental samples are compared with reference samples from a pre-screening used to established the standard curve, since international reference samples exist for anti-CCP only. Patient samples containing high levels of specific antibody were serially diluted in sample buffer to demonstrate the dynamic range of the assay and the upper and lower end of linearity. Activity for each dilution was calculated from the calibration curve using a 4-Parameter-Fit with in-log coordinates. To determine if the curve fit is correct, additional backfit of the standard curve values was done by plotting the standard curve, treating the serial dilution of standards as unknowns and interpolating the values from the standard curve. The readout was close to the expected values (±15%). Additionally, in each run, a positive and negative control were included for comparison with the signal produced by the antibodies.

The cut-off for positivity was determined by receiver operating characteristic (ROC) curve analysis as described in the next section. The decision to use the same cut-off value for all AAPA assays was made practicable by the use of individual optimization of the assays, *via* coating buffers, coating temperature, and antigen concentration. The final performance was verified with the appropriate controls consisting in analysis for distribution of antibody levels in healthy controls (*n* = 191).

In all RA patients, RF and ACPA were measured routinely by nephelometry or the anti-CCP2 assay (Thermo Fisher Scientific, Waltham, MA, USA), respectively.

### Statistical analyses

Descriptive statistics on antibody levels in all cohorts and clinical and descriptive variables of eRA and estRA have been generated. In a first step, ROC curves using antibodies (abs) against ac-lysine, ac-lysine.inv, and ac-ornithine were generated by testing eRA and estRA *versus* healthy subjects to evaluate a cut-off for positivity of AAPA. Prevalence of number of positive peptides per cohort was determined and illustrated. GPA, polymyositis, SLE, and Sjögren patients were summarized as one group [other inflammatory rheumatic diseases (OIRD)] for ROC analyses against eRA. Further ROC curves analyses tested the diagnostic accuracy contrasting eRA and SpA as well as eRA and OIRD. The overlap of the three different AAPA has been evaluated *via* cross-tabulation separately for all cohorts. Venn diagrams have been drawn outlining the overlap of AAPAs and the overlap of AAPA with RF and ACPA status. AAPA in seronegative eRA patients have been evaluated separately. By means of linear regression non-parametric tests and correlation analyses, we evaluated differences in clinical characteristics depending on AAPA positivity and titer. AAPA titers have been illustrated by number of positive AAPA. All analyses were performed using SPSS version 25, Medcalc and STATA.

## Results

### Diagnostic performance of AAPA testing in patients with RA

To establish the diagnostic value of AAPA in patients with eRA, sera from 99 healthy donors and 120 eRA patients (75% female) with a median symptom duration of 0.7 [interquartile range (IQR): 0.3–1.9] years and a median SDAI of 15 (IQR: 9–22) were used for validation of the three assays.^10^ Among the eRA patients, 50% were positive for ACPA and 53.3% for RF, with 46% being positive for both antibodies. In none of the patients were antibodies against the unmodified peptide detected. More details on descriptive statistics are displayed in Table 1.

**Table 1.** Antibody status, clinical and descriptive characteristics of early RA patients (75% female) at baseline separately shown for the total cohort (*n* = 120), AAPA-positive (*n* = 72), AAPA-negative patients, and by number of positive AAPA. Values shown are median (IQR), unless indicated otherwise.

Antibody	Total	AAPA−	AAPA+	1 AAPA	2 AAPA	3 AAPA
*n*	120	48	72	28	32	12
Anti-ac-lysine vimentin (%)	32.5	–	54.2	7.1	78.1	100
Anti-ac-lysine.inv vimentin (%)	35.0	–	58.3	53.6	46.9	100
Anti-ac-ornithine vimentin (%)	39.2	–	65.3	39.3	75.0	100
AAPA total (%)	60.0	–	100	–	–	–
Anti-citrullinated vimentin (%)	44.2	8.3	68.1	39.3	81.3	100
Anti carbamylated vimentin (%)	47.5	12.5	70.8	39.3	90.6	91.7
RF (%)	53.3	31.3	68.1	42.9	84.4	83.3
ACPA (%)	50.0	22.9	68.1	35.7	90.6	83.3
Age (years)	55 (42.7–65)	57 (41.5–65)	54.1 (44.4–63.5)	55.5 (42–65)	53.3 (48.1–61)	51.1 (42.1–61.8)
Duration of disease (years)	0 (−0.2–0.1)	−0.1 (−0.2–0.1)	0 (−0.1–0.1)	−0.1 (−0.4–0.1)	0 (−0.1–0.2)	0 (0–0.3)
Duration of symptoms (years)	0.7 (0.3–1.9)	0.7 (0.3–1.4)	0.9 (0.3–2.1)	0.7 (0.2–1.3)	1.1 (0.5–2.7)	0.8 (0.4–2.1)
C-reactive protein (mg/dl)	0.8 (0.3–1.6)	0.5 (0.2–1.2)	1 (0.4–1.7)	1 (0.5–1.5)	1.2 (0.5–1.9)	1 (0.1–1.9)
Patient global assessment (mm)	36 (17–55.5)	39 (20–54)	33 (15–56.5)	29 (15–52)	40 (14–60)	46 (17–53)
Evaluator global Assessment (mm)	20 (11–30)	18 (11–30)	23 (10.5–31)	23 (13.5–41.5)	23 (11.5–29)	14 (4–30)
Pain (visual analogue scale) (mm)	36.5 (16.3–56.8)	35 (15–58)	40 (16.5–56)	30 (10.5–52)	40 (16.5–62)	42 (18–51)
Swollen joint count – 28 joints	3 (2–5)	3 (2–4)	4 (2–6)	4 (2–6)	4 (2–6)	3 (2–8)
Tender joint count – 28 joints	3 (1–7)	4 (1–8)	2 (1–7)	2 (0.5–4.5)	3 (1–6)	6 (2–17)
SDAI	15.4 (9.1–22.4)	13.4 (9.3–23.3)	16.2 (8.4–22)	13.6 (7.3–23.2)	16.2 (7.5–21.1)	18.4 (12.3–26)
CDAI	14.2 (7.7–20.5)	12.7 (9–22.9)	15 (7.3–20.5)	12.6 (4.9–22.3)	15.3 (7.3–18.7)	17 (8.9–25.7)

AAPA, anti-acetylated peptide antibodies; ACPA, anti-citrullinated-peptide antibodies; CDAI, clinical disease activity index; IQR, interquartile range; SDAI, simplified disease activity index.

**Figure 1.**
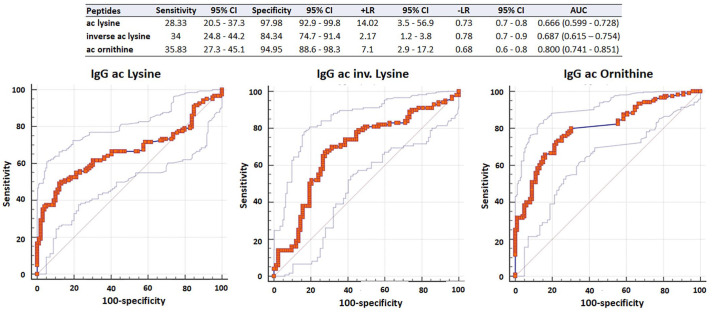
ROC of antibodies against lysine, acetylated lysine.inv, and ornithine in early RA patients *versus* healthy controls. The table depicts AUC (95% CI) of the ROC and sensitivity and specificity at the cut-off at 20 U/ml. AUC, area under the curve; CI, confidence interval; RA, rheumatoid arthritis; ROC, receiver operating curves

**Figure 2.**
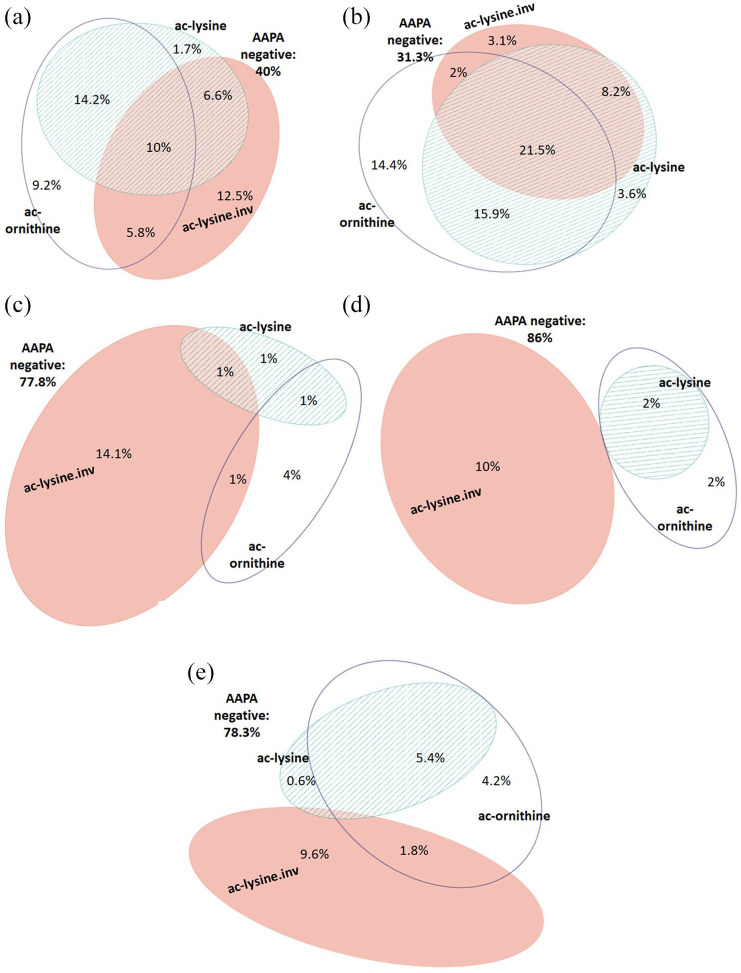
Venn diagram showing the overlap of antibodies against ac-lysine, ac-lysine.inv, and ac-ornithine in patients with rheumatic diseases and healthy subjects indicating the respective percentages of patients testing positive for individual AAPA and combinations thereof. (a) Early RA (*n* = 120). (b) Established RA (*n* = 195). (c) Healthy controls (*n* = 99). (d) Spondyloarthritis (*n* = 50). (e) Other inflammatory rheumatic diseases (SLE, *n* = 49; pSS, *n* = 88; GPA = 14; myositis, *n* = 15). AAPA, anti-acetylated peptide antibodies; GPA, granulomatosis with polyangiitis; pSS, primary Sjögren’s syndrome; RA, rheumatoid arthritis; SLE, systemic lupus erythematosus.

**Figure 3.**
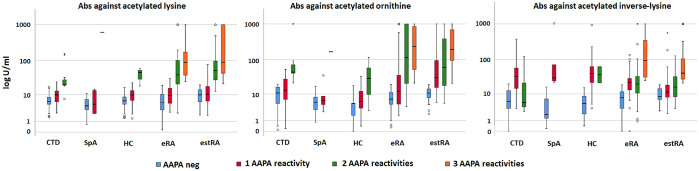
Boxplots showing levels of antibodies to ac-lysine, ac-lysine.inv, and ac-ornithine according to number of AAPA reactivities separately for CTD (connective tissues diseases), SpA, HC, eRA, and estRA. AAPA, anti-acetylated peptide antibodies; CTD, connective tissue diseases; eRA, early RA; estRA, established RA; HC, healthy controls; RA, rheumatoid arthritis; SpA, spondyloarthritis.

To determine appropriate cut-off values, ROC analyses were performed, which revealed different performances of the acetylated peptides: the area under curve (AUC) values of ac-lysine, ac-lysine.inv, and ac-ornithine were 0.666, 0.687, and 0.800, respectively (Figure 1). For further analysis, we decided to use a uniform cut-off of 20 U/ml. At this cut-off, the three peptides showed comparable sensitivities but different specificities (Figure 1, table insert): ac-lysine was the most specific peptide [specificity (spec): 98.0%; sensitivity (sens): 28.3%; positive likelihood ratio (+LR) 14.0], followed by ac-ornithine (spec: 95.0%; sens: 35.8%; +LR 7.1) and ac-lysine.inv, which was the least specific peptide (spec: 84.3%; sens: 34%; +LR 2.2). Altogether, 72 eRA patients (60%) and 22 healthy controls (22%) tested positive for any of the three AAPA (Table 1). Among eRA patients, 44 patients showed multiple reactivities: 32 patients showed two abs (26.6%), which were most frequently directed against ac-lysine and ac-ornithine, and 12 patients (10.0%) showed abs against all three peptides (Figure 2a). Single positivity was detected in 28 patients: 15 patients were positive for ac-lysine.inv, 11 for ac-ornithine, and only two for ac-lysine. Generally, AAPA levels increased with the number of positive abs and were highest in triple positive patients (*p* ⩽ 0.001; Figure 3).

We then tested if multiple antibody reactivities coincided with better diagnostic accuracy. The likelihood of true positive eRA increased with the number of abs. The presence of two or three abs had a sensitivity of 36.7%, a specificity of 97% resulting in a LR+ of 12.1, while triple positivity proved 100% specific for eRA (Table 2).

**Table 2.** ROC curve statistics showing sensitivity, specificity, +LR/−LR for one, two, or three AAPA reactivities in different scenarios (eRA *versus* healthy controls, estRA *versus* healthy controls, eRA *versus* SpA, eRA *versus* OIRD (namely SLE, pSS, myositis, GPA), RF/ACPA negative eRA *versus* healthy, RF/ACPA negative eRA *versus* SpA, RF/ACPA negative eRA *versus* OIRD), including AUC values.

Early RA *versus* healthy						AUC 0.722 (95% CI: 0.658–0.780)		
Number of AAPA	Sensitivity	95% CI	Specificity	95% CI	+LR	95% CI	−LR	95% CI
⩾1	60	50.7–68.8	77.8	68.3–85.5	2.7	1.8–4.0	0.51	0.4–0.7
⩾2	36. 7	28.1–45.9	97.0	91.4–99.4	12.1	3.9–37.8	0.65	0.6–0.8
3	10	5.3–16.8	100	96.3–100.0			0.9	0.8–1.0
Established RA *versus* healthy						AUC: 0.778 (95% CI: 0.726–0.824)		
Number of AAPA	Sensitivity	95% CI	Specificity	95% CI	+LR	95% CI	−LR	95% CI
⩾1	68.7	61.7–75.2	77.8	68.3–85.5	3.1	2.1–4.5	0.4	0.3–0.5
⩾2	47.7	40.5–54.9	97.0	91.4–99.4	15.7	5.1–48.4	0.54	0.5–0.6
3	21.5	16.0–28.0	100	96.3–100.0			0.78	0.7–0.8
Early RA *versus* SpA						AUC: 0.751 (95% CI: 0.679–0.814)		
Number of AAPA	Sensitivity	95% CI	Specificity	95% CI	+LR	95% CI	−LR	95% CI
⩾1	60.00	50.7–68.8	86.00	73.3–94.2	4.3	2.1–8.7	0.47	0.4–0.6
⩾2	36.67	28.1–45.9	98.00	89.4–99.9	18.3	2.6–129.4	0.65	0.6–0.7
3	10.00	5.3–16.8	100.00	92.9–100.0			0.90	0.8–1.0
Early RA *versus* OIRD						AUC 0.713 (95% CI: 0.657–0.765)		
Number of AAPA	Sensitivity	95% CI	Specificity	95% CI	+LR	95% CI	−LR	95% CI
⩾1	60.00	50.7–68.8	78.3	71.3–84.3	2.8	2.0–3.8	0.51	0.4–0.6
⩾2	36.67	28.1–45.9	92.8	87.7–96.2	5.07	2.8–9.2	0.68	0.6–0.8
3	10.00	5.3–16.8	100.00	97.8–100.0			0.90	0.8–1.0
E. RF/ACPA neg early RA *versus* healthy						AUC: 0.599 (95% CI: 0.511–0.678)		
Number of AAPA	Sensitivity	95% CI	Specificity	95% CI	+LR	95% CI	−LR	95% CI
⩾1	40.82	27.0–55.8	77.8	68.3–85.5	1.8	1.1–3.0	0.76	0.6–1.0
⩾2	10.2	3.4–22.2	97.0	91.4–99.4	3.4	0.8–13.5	0.93	0.8–1.0
3	4.08	0.5–14.0	100	96.3–100.0			0.96	0.9–1.0
RF/ACPA neg early RA *versus* SpA						AUC: 0.629 (95% CI: 0.528–0.723)		
Number of AAPA	Sensitivity	95% CI	Specificity	95% CI	+LR	95% CI	−LR	95% CI
⩾1	39.22	25.8–53.9	86.00	73.3–94.2	2.80	1.3–6.0	0.71	0.6–0.9
⩾2	9.80	3.3–21.4	98.00	89.4–99.9	4.90	0.6–40.5	0.92	0.8–1.0
3	3.92	0.5–13.5	100.00	92.9–100.0			0.96	0.9–1.0
RF/ACPA neg early RA *versus* OIRD						AUC: 0.586 (95% CI: 0.517–0.652)		
Number of AAPA	Sensitivity	95% CI	Specificity	95% CI	+LR	95% CI	−LR	95% CI
⩾1	30.6	25.8–53.9	78.31	71.3–84.3	1.81	1.2–2.8	0.78	0.6–1.0
⩾2	6.1	3.3–21.4	92.77	87.7–96.2	1.36	0.5–3.7	0.97	0.9–1.1
3	4.1	0.5–13.5	100.00	97.8–100.0			0.96	0.9–1.0

AAPA, anti-acetylated peptide antibodies; ACPA, anti-citrullinated-peptide antibodies; AUC, area under the curve; CI, confidence interval; eRA, early RA; estRA, established RA; GPA, granulomatosis with polyangiitis; HC, healthy controls; +LR, positive likelihood ratio; –LR, negative likelihood ratio; OIRD, other inflammatory rheumatic diseases; pSS, primary Sjögren’s syndrome; RA, rheumatoid arthritis; RF, rheumatoid factor; ROC, receiver operating curves; SLE, systemic lupus erythematosus; SpA, spondyloarthritis

Comparing AAPA positive *versus* AAPA negative eRA patients, disease activity and individual core set variables were similar in the two groups, except that AAPA-positive patients had a higher likelihood to also be RF and/or ACPA positive (*p* < 0.001) (Table 1). Furthermore, patients with multiple AAPA reactivities seemed to have longer symptom duration but this did not reach the level of statistical significance (*p* = 0.074). In patients negative for RF and ACPA (seronegative), we found no differences in any of these variables between AAPA positive and AAPA negative patients at baseline.

**Figure 4.**
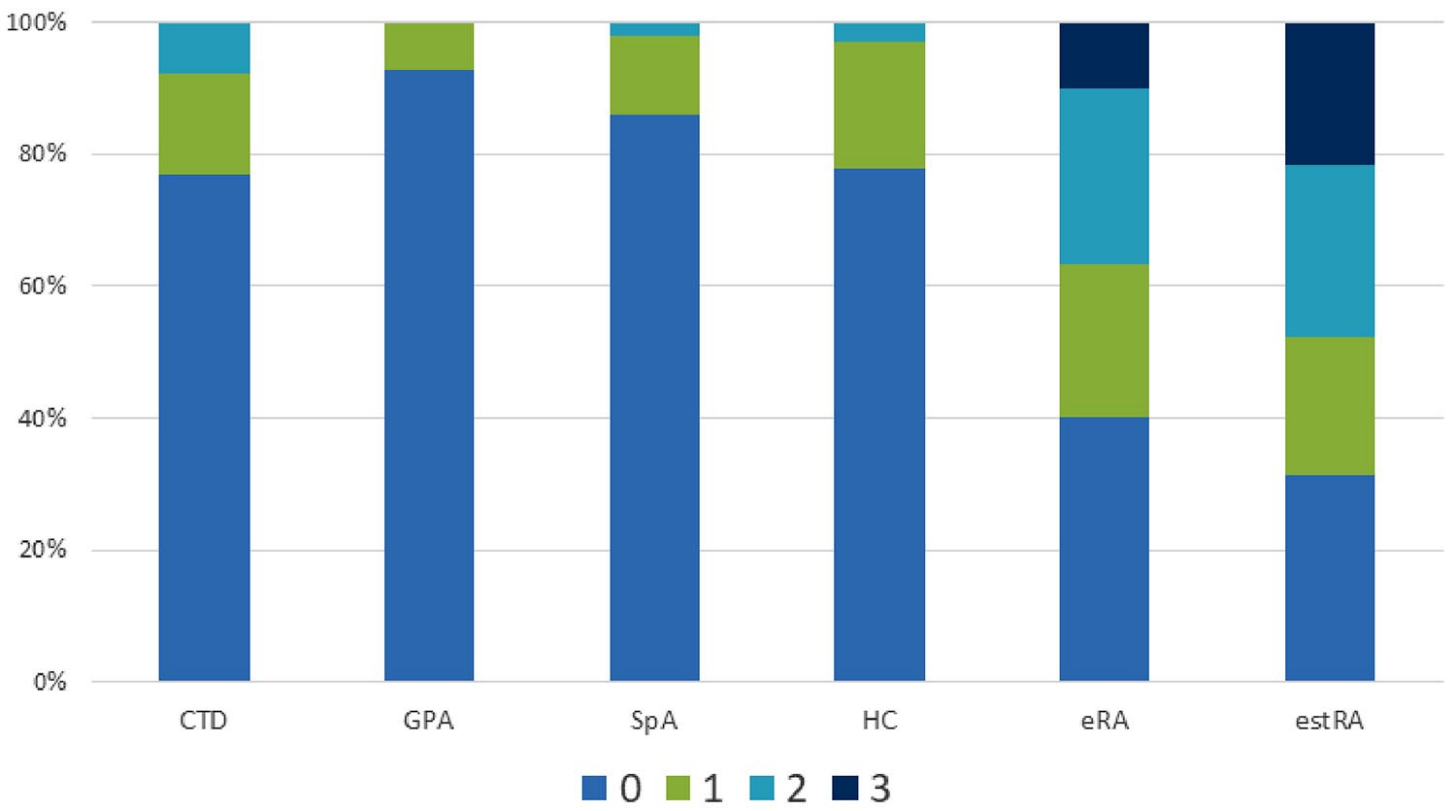
Distribution of numbers of AAPA reactivities in rheumatic diseases and healthy subjects. Percentages of patients showing 0, 1, 2, or 3 AAPA reactivities are indicated. CTD (polymyositis, *n* = 15; SLE, *n* = 49; pSS, *n* = 88); GPA, *n* = 14, SpA, *n* = 50; HC, *n* = 99; eRA, *n* = 120; estRA, *n* = 195. AAPA, anti-acetylated peptide antibodies; CTD, connective tissue diseases; eRA, early RA; estRA, established RA; GPA, granulomatosis with polyangiitis; HC, healthy controls; pSS, primary Sjögren’s syndrome; RA, rheumatoid arthritis; SLE, systemic lupus erythematosus; SpA, spondyloarthritis.

To substantiate and validate the data obtained in patients with early untreated RA, we analyzed AAPA additionally in a cohort of 195 patients with estRA (79% female, median symptom duration: 6.6 years (2.4–13) years, 57% positive for ACPA and 59% for RF). This analysis revealed an AAPA testing sensitivity of 68.7%, which was somewhat higher than in eRA (Table 2). Interestingly, the percentage of patients triple positive for AAPA doubled in comparison with eRA with 21.5% of the estRA patients showing all three abs and 26.1% being double positive (Figures 2b and 4). Thus, nearly 50% of the estRA patients showed multiple reactivities resulting in a LR+ of 15.7 (Table 2).

### Prevalence of AAPA across other rheumatic diseases

We next determined the prevalence of AAPA in patients with other rheumatic diseases, including SpA (*n* = 50), SLE (*n* = 49), pSS (*n* = 88), IIM (*n* = 15), and GPA (*n* = 14). Prevalence of AAPA was generally lower than in RA, and, in the majority of cases, only one of the three peptides was recognized, preferentially ac-lysine.inv, which showed a similar prevalence as in HC (Figure 2d, 4e). Especially when considering differential serodiagnostics, AAPA showed a test performance for differentiating eRA *versus* SpA similar to eRA *versus* HC (AUC: 0.751; Table 2). Remarkably, double reactivity was seen in only one SpA patient (LR+ 18.3) and triple reactivity was not observed at all (Table 2). Among the three peptides ac-lysine and ac-ornithine proved highly specific for RA, showing LR+ of 15.8 and 9.6, respectively (Table 3).

**Table 3.** Diagnostic test statistics of individual AAPA to discriminate eRA *versus* SpA: sensitivity, specificity and likelihood ratios.

AAPA	Sensitivity	95% CI	Specificity	95% CI	+LR	95% CI	−LR	95% CI
*Ac-lysine*	32.50	24.2–41.7	98.00	89.4–99.9	16.25	2.3–115.1	0.69	0.6–0.8
*Ac-lysine.inv*	42.00	32.2–52.3	82.76	64.2–94.2	2.44	1.1–5.6	0.70	0.6–0.9
*Ac-ornithine*	39.17	30.4–48.5	96.00	86.3–99.5	9.79	2.5–38.8	0.63	0.5–0.7

AAPA, anti-acetylated peptide antibodies; CI, confidence interval; eRA, early RA; +LR, positive likelihood ratio; –LR, negative likelihood ratio; SpA, spondyloarthritis.

The test-performance of AAPA for differentiating eRA from OIRD had similar sensitivity and specificity with an AUC of 0.713 as had been found for eRA *versus* HC (Table 2). Double reactivities were observed in 7% of OIRD patients (Figure 2e) and were preferentially directed to ac-lysine and ac-ornithine. Double reactivity against ac-lysine and ac-lysine.inv, as well as triple reactivity, was not observed and therefore proved again highly specific for RA (Figure 2e). Abs against ac-lysine showed the highest discriminatory capacity for RA *versus* OIRD (LR+: 5.4 CI%95: 2.8–10.4) (Table 4). Taken together, ac-lysine appeared to have the highest diagnostic value among the three peptides to discriminate RA from SpA and OIRD; double reactivity in any combination proved equally potent, and triple reactivity was by far the best discriminator, being observed exclusively in RA patients. These data are summarized in Figure 4. Furthermore, as already observed in RA patients ab levels increased in patients with OIRD by numbers of AAPA reactivities (*p* = 0.023). Moreover, AAPA levels were higher in eRA than in OIRD (all *p* < 0.005) (Figure 3).

**Table 4.** Diagnostic test statistics of AAPA to discriminate eRA *versus* OIRD: Sensitivity, specificity and likelihood ratios.

AAPA	Sensitivity	95% CI	Specificity	95% CI	+LR	95% CI	−LR	95% CI
*Ac-lysine*	32.50	24.2–41.7	93.98	89.2–97.1	5.40	2.8–10.4	0.72	0.6–0.8
*Ac-lysine.inv*	42.00	32.2–52.3	86.52	79.8–91.7	3.12	1.9–5.0	0.67	0.6–0.8
*Ac-ornithine*	39.17	30.4–48.5	88.55	82.7–93.0	3.42	2.1–5.5	0.69	0.6–0.8

AAPA, anti-acetylated peptide antibodies; CI, confidence interval; eRA, early RA; +LR, positive likelihood ratio; –LR, negative likelihood ratio; OIRD, other inflammatory rheumatic diseases.

### AAPA in relation to RF and ACPA in patients with eRA

**Figure 5.**
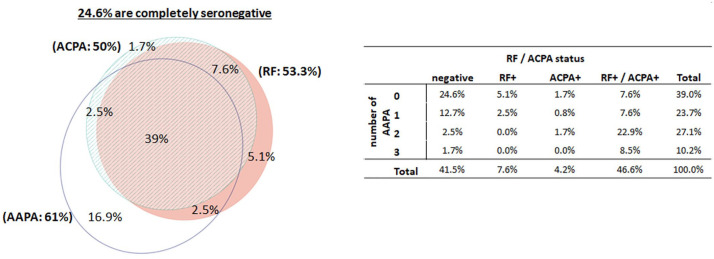
Venn diagram, outlining the overlap between AAPA, RF and ACPA in patients with early RA, indicating the percentages of patients testing positive for the three antibodies and combinations thereof. The accompanying cross-table outlines the correlations between AAPA and RF/ACPA status; 24.6% of early RA patients show neither AAPA nor RF nor ACPA. AAPA, anti-acetylated peptide antibodies; ACPA, anti-citrullinated-peptide antibodies; eRA, early RA; RA, rheumatoid arthritis; RF, rheumatoid factor.

Seropositivity for ACPA and/or RF was seen in 58% of eRA patients: 39% were triple positive for AAPA/RF/ACPA, 2.5% were double positive for AAPA/RF, and another 2.5% were double positive for AAPA/ACPA (Figure 5). Within the AAPA/RF/ACPA-positive patients, abs against ac-ornithine were the most common single AAPA reactivity (15.2%), if only one of the AAPA was detectable. In 35% of AAPA/RF/ACPA-positive patients, AAPA were directed against ac-ornithine and ac-lysine, and 22% showed all AAPAs. Most of the remaining patients were double positive for ac-lysine.inv and ac-lysine or ac-ornithine. Thus, the most frequent AAPA reactivity was directed against ac-ornithine, which was targeted in 85% of the AAPA/RF/ACPA positive patients. Importantly, 17% of eRA patients were found to be exclusively AAPA positive, meaning that approximately 40% of the seronegative patients showed reactivities against acetylated peptides. Among these patients, 15 showed only one AAPA while three of the patients showed two reactivities and two were triple positive (Figure 5, table insert). For comparison, six and two of the eRA patients were solely positive for RF or ACPA, respectively. In seronegative patients, the presence of one AAPA identified RA patients *versus* healthy subjects with a specificity of 78% and those with two AAPA reactivities with 97% specificity (Table 2). To demonstrate the added diagnostic value of AAPA in “seronegative” patients, we were interested to see the prevalence of abs to the citrullinated and carbamylated isoforms of the vimentin peptide. Antibodies to carb-vimentin were seen in seven patients (14%) and abs to cit-vimentin in four (8%) patients. Importantly, among the 20 AAPA positive patients, abs against cit-vimentin could be detected in 3 patients and abs to the carbamylated peptide were found in 4 patients. Considering that in 1 of the 20 patients these two abs overlapped, 14 patients remained solely AAPA-positive, further narrowing the diagnostic gap of seronegativity.

Abs against ac-lysine.inv was the most prevalent reactivity, being detected in almost 35% of seronegative eRA patients, compared with 16% in healthy subjects, 10% in SpA, and 11% in OIRD. This translates into a LR+ of 2.2 (95% CI: 1.0–5.8) compared with HC, 2.4 (95% CI: 1.0–5.8) to SpA and 3.1 (95% CI: 1.8–5.4) to OIRD respectively. Double AAPA reactivity showed a specificity well above 90% both against SpA and OIRD (Table 2). Of note, double reactivity against ac-lysine and ac-lysine.inv was observed in 4% of seronegative RA patients but not in any of the 216 patients with other rheumatic diseases, and proved therefore as specific for RA as triple positivity, which was detected in another 4% of seronegative patients.

## Discussion

Autoantibodies to post-translationally modified epitopes are the serological hallmark of RA and considered to be most valuable diagnostic markers. They are now generally termed anti-modified protein antibodies (AMPA) and include ACPA, antibodies to carbamylated epitopes, antibodies to acetylated epitopes, and antibodies to malonaldehyde adducts.^22,32^ However, currently only RF and ACPA are used in routine diagnostics because the added diagnostic value of the other AMPAs is still uncertain and commercial assays are not yet available.

This study provides some novel insights into the diagnostic value of antibodies to acetylated epitopes that have only recently been described to occur in patients with RA.^10,18^ They were found mostly in ACPA positive patients and partially cross-react with ACPA and/or anti-CarP antibodies.^22,33,34^ Nevertheless, AAPA and other AMPAs may also be present in a subgroup of seronegative patients, indicating that they might have added diagnostic value for reducing the serological gap. In the initial study employing a single acetylated peptide (ac-lysine) and involving only patients with early arthritis they seemed to be less specific than ACPA, but so far this issue has not been thoroughly investigated.^18^ In our study, we used three peptides for AAPA detection and investigated their reactivity in patients with early RA, established RA, and an appropriate number of controls including healthy subjects and patients with various inflammatory rheumatic diseases, which were not explored in great detail in previous studies. In contrast to most previous studies, standard curves were used and cut-offs determined by ROC curve analysis, allowing us to obtain quantitative results expressed as arbitrary units.

It emerged that AAPA are equally prevalent (60%) as RF (53%) or ACPA (50%), which were measured by nephelometry and an anti-CCP2 assay, respectively. Even without the least specific peptide, ac-lysine.inv, the prevalence of AAPA still would be close to 50%. In eRA, the prevalence of individual AAPA ranged from 32% to 39%, which, for ac-lysine, was in reasonable agreement with data from two previous studies,^18,33^ taking into account that the definition of cut-offs for positivity differed between the three studies. A very recent publication by Rodriguez-Martínez *et al.* demonstrated the value of ac-ornithine implemented in the American College of Rheumatology/*European* League Against Rheumatism (ACR/EULAR) classification criteria for RA.^6^ Their data on sensitivity and specificity of AAPA are in good agreement with our results. However, in this study, a high prevalence of AAPA in RF/ACPA negative patients could not be shown, because only ac-lysine and ac-ornithine antibodies were determined,^33^ while we found ac-lysine.inv to be the most common antibody in RF/ACPA-negative eRA. Furthermore, in the study of Rodriguez-Martínez *et al*., different cut-offs were used for the two assays, whereas we decided to use uniform cut-offs for all three assays, which increased the overall sensitivity of AAPA testing. Thus, we aimed to determine the capacity of individual AAPAs and combinations thereof to discriminate between early (untreated) RA and other inflammatory rheumatic diseases. Apart from the relatively high sensitivity, the most remarkable finding was that the presence of two AAPA increased specificity of AAPA testing considerably. This was also true for combinations with ac-lysine.inv, which showed only moderate specificity for RA when occurring as single reactivity (see below). Importantly, the presence of three AAPA appeared to be 100% specific since triple positivity was not seen in other diseases at all. The high specificity was most pronounced when comparing RA with SpA. While 12% of SpA patients compared with 23% of eRA patients showed single positivities, only 2% of SpA as compared with 36.6% of eRA patients showed multiple AAPA reactivities. Double reactivities were otherwise seen in approximately 10% of SLE and Sjögren’s patients, in 3% of HC.

While as in previous studies, AAPA were detected frequently in seropositive patients. Importantly, also 40% of the RF/ACPA-negative patients were AAPA positive, suggesting that measuring AAPA could be used in addition to RF and ACPA to characterize seropositive early RA. Although most seronegative patients were single positive, still the likelihood to have RA was 1.8 times higher in seronegative eRA showing only one AAPA. However, the presence of two AAPA (detected in 6% of seronegative eRA patients) increased the risk for RA several-fold while the presence of three AAPA (seen in another 4%) was absolutely specific for RA. Thus, multiple reactivity AAPA had a high capacity to discriminate seronegative RA from other rheumatic diseases. When also considering the presence of antibodies to the carbamylated vimentin peptide, it turned out that most AAPA positive patients (80%) were negative for anti-carP antibodies, which were detected in only 14% of seronegative patients. Thus, AAPA, together with anti-carP antibodies, may indeed contribute to closing the gap of seronegativity left by the routinely used RF and anti-CCP2 assays.

Considering single reactivities, ac-lysine was the most specific antigen, followed by ac-ornithine (+LR of ac-lysine and ac-ornithine *versus* SpA and OIRD were 16.3; 9.8 and 5.4; 3.4, respectively) whereas ac-lysine.inv was clearly the least specific, occurring with comparable prevalence (7–16%) in disease controls and healthy subjects. However, combinations of anti-ac-lysine.inv with one of the other two AAPA reactivities proved very specific for RA, being detectable in 12.5% of eRA patients (and in 10.3% of patients with established disease) but in only 2% of disease controls and healthy subjects. For comparison, dual reactivity against ac-lysine and ac-ornithine was seen in 14.2% of eRA, 4.5% of disease controls, and 1% of healthy subjects.

While the mechanisms leading to the generation of AAPAs need to be further characterized, it is interesting to note that lysine acetylation has been linked to the gut microbiota. Germ-free mice colonised with microbiota from conventionally reared mouse donors exhibited a dramatically different pattern of lysine acetylation after colonization.^35^ The links between the oral and gut flora and the disease status in RA are increasingly coming to light and it is tempting to speculate that microbiota-associated lysine acetylation represents one of the mechanisms establishing that link.^36,37^

Of note, the most prevalent AAPA reactivity was directed to ac-ornithine in our study, as well as in previous studies.^10,32^ Ornithine-like citrulline is an unusual amino acid that is not encoded in the genome but created by non-enzymatic post-translational modification as described by several authors.^38,39^ Interestingly however, this does not appear sufficient to create a neo-epitope since only the peptide carrying acetylated ornithine was targeted by autoantibodies. Thus, it would be tempting to speculate that ac-ornithine containing neo-epitopes generated by aberrant post-translational modification form one of the primary targets of the AAPA response, which then may partially cross-react with ac-lysine containing epitopes but also with citrullinated or carbamylated epitopes.^34,40,41^ It is also not clear which, among the plethora of acetylated, citrullinated, or carbamylated proteins, is the primary target of this autoimmune response – a question currently under scrupulous investigation.

Due to the relatively small number of seronegative patients, we could not yet investigate whether AAPA positive patients shows a clinical picture similar to seropositive RA in terms of disease progression and outcome. We expect that at least patients with multiple reactivities may be similar to seropositive patients, also because AAPA titers were significantly higher than in single-positive RA patients or single-positive controls. In line with this assumption, RF/anti-CCP2 seronegative RA patients showing multiple reactivities against various citrullinated peptides were found to be clinically similar to seropositive patients.^42–44^ Also, the very high predictive value for RA of detecting RF/ACPA/anti-CarP triple positivity in early arthritis patients fits in well into this picture.^45^ Therefore, measuring AAPA could be used to confirm the diagnosis of RA in the absence of RF and ACPA, reducing the serological gap of seronegativity and/or to improve classification of RA as suggested recently by Rodriguez-Martinez *et al*.^33^ Once assays for AAPA and other AMPAs become generally available, preferably multiparameter assays measuring multiple antibodies by array or Luminex technology, these issues could be further investigated. Prospective longitudinal studies could then be performed, which may open new avenues for personalized medicine decision making.
